# Protein Tyrosine and Serine/Threonine Phosphorylation in Oral Bacterial Dysbiosis and Bacteria-Host Interaction

**DOI:** 10.3389/fcimb.2021.814659

**Published:** 2022-01-11

**Authors:** Liang Ren, Daonan Shen, Chengcheng Liu, Yi Ding

**Affiliations:** State Key Laboratory of Oral Diseases, National Clinical Research Center for Oral Diseases, West China Hospital of Stomatology, Sichuan University, Chengdu, China

**Keywords:** oral bacteria, kinase, phosphatase, tyrosine phosphorylation, serine phosphorylation, bacterial dysbiosis

## Abstract

The human oral cavity harbors approximately 1,000 microbial species, and dysbiosis of the microflora and imbalanced microbiota-host interactions drive many oral diseases, such as dental caries and periodontal disease. Oral microbiota homeostasis is critical for systemic health. Over the last two decades, bacterial protein phosphorylation systems have been extensively studied, providing mounting evidence of the pivotal role of tyrosine and serine/threonine phosphorylation in oral bacterial dysbiosis and bacteria-host interactions. Ongoing investigations aim to discover novel kinases and phosphatases and to understand the mechanism by which these phosphorylation events regulate the pathogenicity of oral bacteria. Here, we summarize the structures of bacterial tyrosine and serine/threonine kinases and phosphatases and discuss the roles of tyrosine and serine/threonine phosphorylation systems in *Porphyromonas gingivalis* and *Streptococcus mutans*, emphasizing their involvement in bacterial metabolism and virulence, community development, and bacteria-host interactions.

## Introduction

The oral microbiome is the second largest and most diverse microbiota in the human body, encompassing approximately 1,000 species (Lamont et al., 2018). According to the expanded Human Oral Microbiome Database (eHOMD), the oral bacteria are highly diverse, and account for the majority of oral microorganisms, composed mainly of six major phyla: Firmicutes, Bacteroidetes, Proteobacteria, Actinobacteria, Spirochaetes and Fusobacteria (Escapa et al., 2018). In healthy systems, the polymicrobial communities maintain an ecological balance *via* intermicrobial and host microbial interactions. Dysbiosis, or perturbations in the composition of commensal communities, is a driver of the host immune inflammatory response and can disrupt host tissue homeostasis, promoting oral diseases such as dental caries and periodontitis (Lamont et al., 2018; Hajishengallis and Lamont, 2021). Oral bacteria can also directly or indirectly affect a variety of systemic diseases, such as cardiovascular disease and diabetes (Hajishengallis and Chavakis, 2021). Although some controversies remain, several potential mechanisms have been proposed, including (1) bacteria entering the blood circulation, resulting in distant dissemination; (2) systemic injury by free toxins of oral bacteria; (3) stimulation of systemic inflammation by soluble antigens of oral bacteria; and (4) inducing dysbiosis of gut microbiota (Hajishengallis and Chavakis, 2021). Notably, *Porphyromonas gingivalis*, a keystone pathogen in periodontitis, expresses a variety of virulence factors (*e.g.*, lipopolysaccharide, outer membrane vesicles and fimbriae) that facilitate its survival, spreading and disrupting the immune response (Zhang et al., 2020). The colonization of *P. gingivalis* can remodel the commensal bacterial community, thus promoting the bacterial dysbiosis and the imbalance of bacteria-host interactions. The transition from homeostatic balance to dysbiosis and imbalance plays a central role in oral microbial diseases (Lamont et al., 2018; Hajishengallis and Lamont, 2021).

Evidence has shown that post-translational modifications (PTMs) are critical processes used by oral bacteria to modify proteins and coordinate the signaling networks, and are therefore involved in the regulation of bacterial communities and bacteria-host interactions (Whitmore and Lamont, 2012). In fact, protein phosphorylation is a critical covalent protein modification in signal transduction pathways. By combining or separating small molecular phosphates with substrate amino acid residues, phosphates can be passed along these information pathways, causing a cascade of signal transduction protein alterations, thus allowing signal transmission (Low et al., 2021). This process is modulated by two families of enzymes: kinases and phosphatases (Hardie, 1990). Protein kinases and their cognate phosphatases play extensive roles in many basic physiological processes in bacteria, including signal transduction, growth control and malignant transformation, as well as in regulating bacterial pathogenicity and antibiotic resistance (Kyriakis, 2014; Shaban et al., 2020; Shamma et al., 2021). Many studies have emphasized protein phosphorylation which occurs in prokaryotes (Bonne Kohler et al., 2020). Phosphorylation of tyrosine and serine/threonine residues is the most prevalent PTM.

Pioneering investigations of the tyrosine and serine/threonine phosphorylation in bacteria began in the 1970s (Wang and Koshland, 1978; Manai and Cozzone, 1979). In the early 1990s, the first protein kinase PknL was discovered in *Myxococcus xanthus*. This enzyme shares a structural similarity with eukaryotic serine/threonine kinases (STKs) and is required for the normal development of *M. xanthus* (Muñoz-Dorado et al., 1991). Later, the first bacterial phosphatase was discovered by G A Nimmo et al. who reported that isocitrate dehydrogenase (IDH) is regulated by phosphorylation in *Escherichia coli* (Nimmo et al., 1984; Nimmo and Nimmo, 1984). Phosphorylation systems modify bacterial proteomes, imparting cells with rapid and reversible responses to specific environmental stimuli (Janczarek et al., 2018). Evidence has indicated a close association between phosphorylation and bacterial pathogenesis. For instance, *Mycobacterium tuberculosis* can secrete the eukaryotic serine-threonine protein kinase PknG into host macrophages by blocking the transition of Rab7l1-GDP to Rab7l1-GTP in a kinase activity-dependent process, thus realizing its pathogenic potential by facilitating bacterial survival inside human macrophages (Shimizu et al., 1997; Pradhan et al., 2018). PtpA, a tyrosine phosphatase secreted by *Mycobacterium*, can also inhibit the fusion of phagosomes and lysosomes, which helps pathogens to evade host immune mechanisms (Jaiswal et al., 2019). Further evidence has been derived from *Streptococcus pneumoniae*. The tyrosine phosphatase PhpP regulates proteins phosphorylation by direct dephosphorylation of target protein and dephosphorylation of its homologous kinase StkP, thus coordinating cell wall synthesis and division of *S. pneumoniae* (Sasková et al., 2007; Osaki et al., 2009). The *PhpP* mutant of *S. pneumoniae* displayed insufficient cell elongation and increased sensitivity at high temperature and oxidative stress, as well as decreased genetic transformation ability (Ulrych et al., 2016).

Bacterial protein kinases and phosphatases are closely interconnected, regulating phosphate transmission and covalent modifications, and contributing to bacterial pathogenesis. However, the reciprocal relationships between oral bacterial protein tyrosine and serine/threonine phosphorylation and pathogenesis remain to be elucidated. This review focuses on two well-known oral pathogens, *Streptococcus mutans* and *P. gingivalis*, aiming to summarize the present knowledge of the structural and functional aspects of kinases and phosphatases in oral bacteria, with emphasis on the role of tyrosine and serine/threonine phosphorylation in oral bacterial dysbiosis and oral bacteria-host interactions.

## The Structure of Bacterial Tyrosine and Serine/threonine Kinases and Phosphatases

When bacteria perceive external stimulation, kinases undergo autophosphorylation and catalyze the phosphorylation, *i.e.* the transfer of the γ-phosphate group from nucleoside triphosphates, usually adenosine triphosphate (ATP) to other proteins (Pereira et al., 2011). Bacterial phosphatases remove the covalently linked phosphate group from the phosphorylated protein (phosphoprotein) by hydrolysis (dephosphorylation), thereby maintaining the stability of the physiological environment. Kinases and phosphatases act as switches to regulate specific signal transduction pathways (Huse and Kuriyan, 2002). In bacteria, protein kinases can be classified into five types: histidine kinases (His kinases), tyrosine kinases (Tyr kinases), arginine kinases (Arg kinases), Hanks-type Ser/Thr kinases (STKs) (commonly known as eukaryotic-like STKs), and atypical serine kinases (Janczarek et al., 2018). Among them, Tyr kinases and STKs can phosphorylate various proteins and regulate bacterial physiology (Mijakovic et al., 2016). Compared to kinases, fewer bacterial phosphatases have been discovered and biochemically characterized. The protein phosphatase family in bacteria can be divided into four categories: phosphoprotein phosphatases (PPPs), metal-dependent phosphatases (PPMs) acting on serine/threonine residues, low-molecular-weight protein tyrosine phosphatases (LMW-PTPs), and Asp-based phosphatases (Wright and Ulijasz, 2014; Esser et al., 2016).

### Tyrosine Kinases

**Figure 1:**
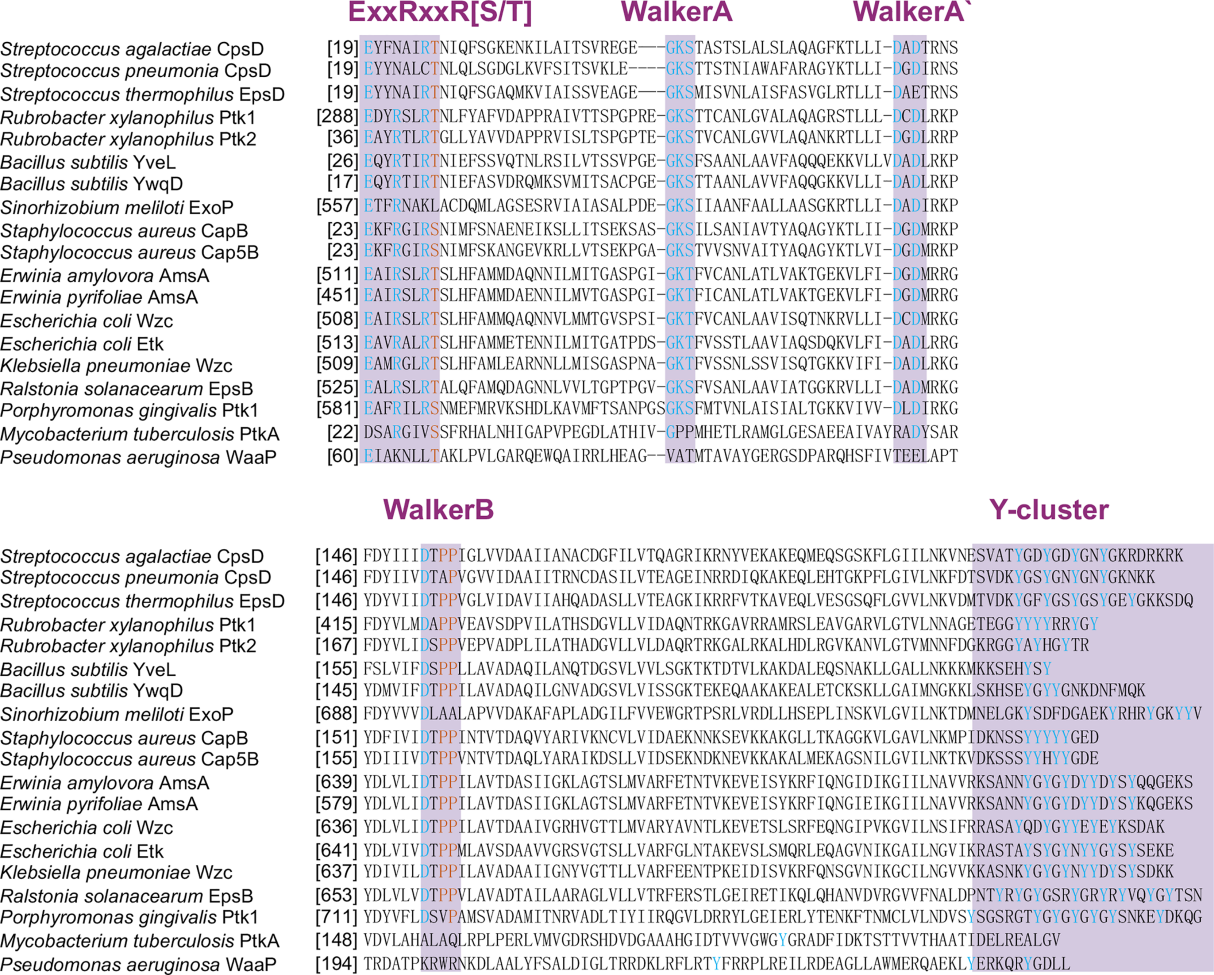
The active motif of BY kinase. Conservative ExxRxxR motif, canonical Walker A motif (GxxxxGK[S/T]), Walker B motif ([ilvfm](3)DxDxR), and a tyrosine-rich cluster (Y cluster) at the C-terminal sites are common features of the BY kinase from 19 bacteria.

**Figure 2:**
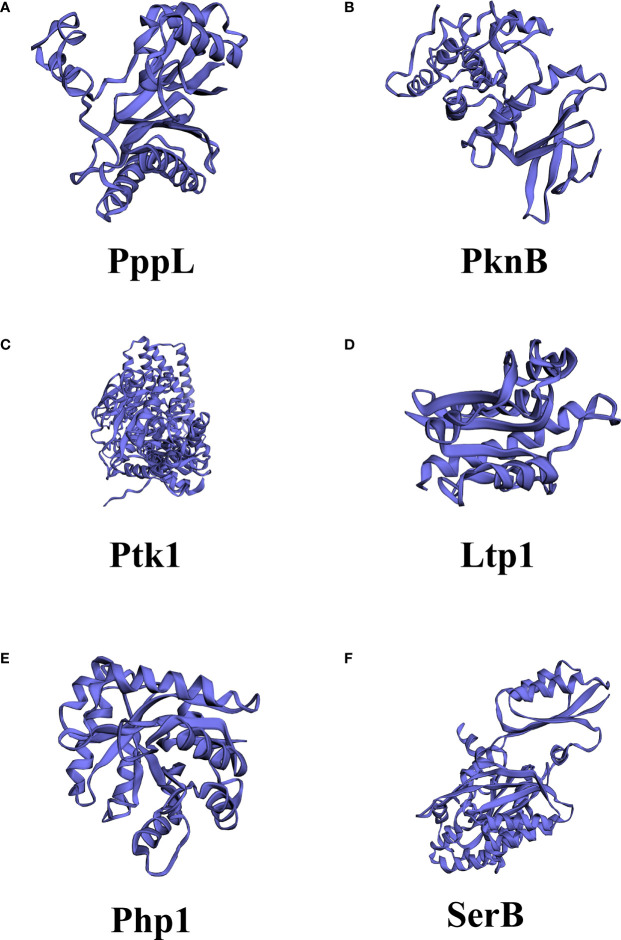
Different structures among kinases and phosphatase in *Streptococcus mutants* and *Porphyromonas gingivalis*. **(A)** PppL*, S. mutants* PPM family protein phosphatase; **(B)** PknB*, S. mutants* serine/threonine protein kinase; **(C)** Ptk1, *P. gingivalis* tyrosine kinase; **(D)** Ltp1*, P. gingivalis* low molecular weight protein-tyrosine phosphatase; **(E)** Php1*, P. gingivalis* PHP family tyrosine phosphatase **(F)** SerB*, P. gingivalis* Serine/threonine protein phosphatase. The structures were predicted by PHYRE2 Protein Fold Recognition Server.

**Figure 3:**
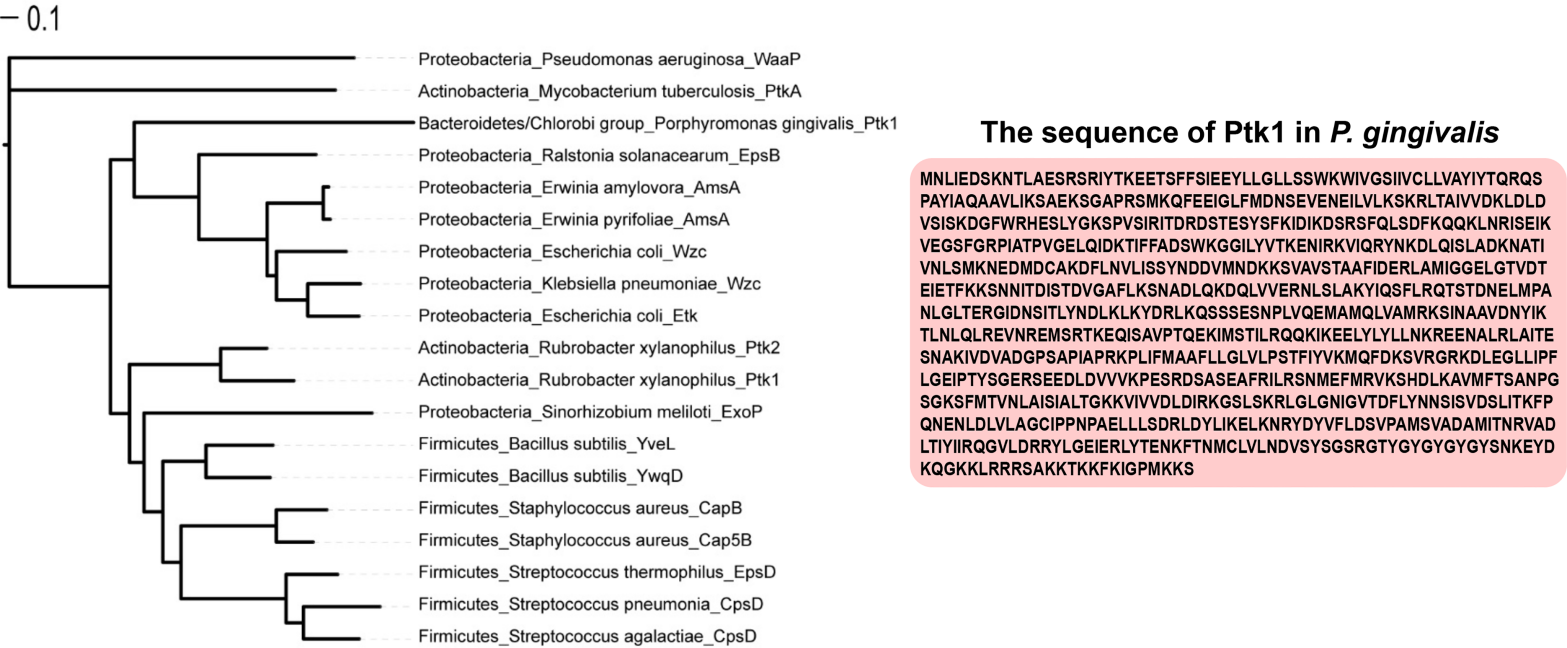
Phylogenetic tree analysis of bacterial tyrosine kinase. Phylogenetic tree analysis of 19 bacterial BY kinases by the RAxML maximum likelihood method and visualization of the results with iTOL v6.

Protein phosphorylation on tyrosine residues is catalyzed by autophosphorylating ATP-dependent tyrosine kinases that exhibit structural and functional features similar to those of their eukaryotic counterparts. Most enzymes discovered in bacteria with tyrosine kinase activity discovered in bacterial are bacterial tyrosine kinases (BY kinases). The structure of BY kinases has been comprehensively reviewed (Whitmore and Lamont, 2012). In brief, BY kinases have a transmembrane domain and an intracellular catalytic domain (Doublet et al., 2002). The transmembrane domain interacts with other proteins through the outer membrane and affects the cellular function of tyrosine kinase, which is critical for triggering kinase activity (Collins et al., 2006). The conservative ExxRxxR motif, canonical Walker A motif (GxxxxGK[S/T]), Walker B motif ([ilvfm](3)DxDxR), and a tyrosine-rich cluster (Y cluster) at the C-terminal sites are common features of the BY-kinase family (Grangeasse et al., 2007). Some BY kinases have an additional Walker A’ motif [(ILVFM(3)DxxP)] (**Figure 1**). BY kinases autophosphorylate in the Y clusters to facilitate their interaction with other proteins. The steps of signal transduction in BY-kinases are similar to those in the eukaryotic signal transduction cascade. For instance, the Tyr (569) residue of Wzc, a BY kinase of *Escherichia coli* K12, can autophosphorylate, resulting in an increased protein kinase activity (Grangeasse et al., 2002).The phosphorylation level in the tyrosine-rich cluster may affect the intensity of the interaction between BY-kinase and other proteins (Collins et al., 2006). Ptk1, which is the first discovered BY kinase in *P. gingivalis*, contains ExxRxxR, Walker A, Walker A’, Walker B motifs, and a C-terminal Y cluster (Wright et al., 2014) (**Figures 1**, **2**). All of these domains are required for kinase autophosphorylation and substrate phosphorylation activity. And Ptk1 is highly homologous to Wzc *Escherichia coli* (**Figure 3**). Moreover, the functional phosphor-transfer is indispensable for Ptk1-mediated control of *P. Porphyromonas gingivalis-Streptococcus gordonii* community formation and extracellular polysaccharide biosynthesis (Liu et al., 2017). The ubiquitous bacterial kinase (UbK) family is a newly discovered tyrosine kinase family in oral bacteria. The UbK family was originally classified as an unknown but essential P-loop ATPase (Karst et al., 2009). A recent study revealed that the UbK family members can auto-phosphorylate and phosphorylate protein substrates on S/T and Y residues, which classifies them as dual-specific kinases (Nguyen et al., 2017). Structurally, UbK contains a conserved domain: the Walker A motif, HxDxYR, SPT/S and EW motifs. Ubk1 is a UbK family member in *P. gingivalis* that can autophosphorylate on the tyrosine and serine residues within the HxDxYR and SPT/S domains, respectively (Perpich et al., 2021).

### Tyrosine Phosphatase

There are three categories of protein tyrosine phosphatases: eukaryotic like phosphatases (PTPs) and dual-specific phosphatases; low molecular weight protein tyrosine phosphatases (LMW-PTPs), and the less common polymerase–histidinol phosphatases (PHPs), which are often found in gram-positive bacteria (Whitmore and Lamont, 2012). Some LMW-PTPs are similar to eukaryotic low molecular weight peptide, and the other part has typical characteristics of prokaryotic LMW-PTPs, such as Wzb in *E. coli* (Lescop et al., 2006). Eukaryotic and prokaryotic LMW-PTPs diverged during the evolution process. For example, there are two tyrosine phosphatases (PtpA and PtpB) in both *Staphylococcus aureus* and *Bacillus subtilis* (Soulat et al., 2002; Xu et al., 2006). PTPs, dual-specific phosphatases and LMW-PTPs utilize a common catalytic mechanism that contains the conserved signature C(x)5R motif, where cysteine and arginine residues are important for the catalytic activity. Functioning as a nucleophile, cysteine attacks the phosphorus atom of the phosphor-tyrosine residue of the substrate, while the arginine residue interacts with the phosphate moiety of the phosphor-tyrosine (Tiganis, 2002). This motif is flanked by an important aspartic acid residue, whose position varies among the families. Unlike the other members in PTPs, PHPs are divalent metal ion-dependent phosphor-tyrosine phosphatases, whose catalytic mechanism is metal-dependent (Kim et al., 2011; Standish and Morona, 2014). PHPs show optimal activity at basic pH and depend on the presence of a metal ion, especially when combined with Mn^2+^ (Mijakovic et al., 2005). This mechanism also requires an arginine residue in the active site and a nucleophilic attack by metal-bound water, even if it is dependent on metal ions (Hagelueken et al., 2009). Recently, a tyrosine phosphatase (Php1) belonging to the PHP family of *P. gingivalis* was reported by Jung et al. (2019). Php1 maintains all the invariant histidine, aspartate, and arginine residues in four conserved motifs, similar to other bacterial PHP-PTP proteins, such as in *M. xanthus* and *S. pneumoniae*, and shows high structural conservation with YwqE, a PHP-PTP in *B. subtilis* (Jung et al., 2019).

### Serine/Threonine Kinases

The first structurally characterized bacterial serine/threonine kinase was PknB in *M. tuberculosis*, which revealed a striking similarity of a two-lobe structure to the eukaryotic versions in terms of its two-lobe structure (Ortiz-Lombardía et al., 2003). The two-lobe structure of serine/threonine kinase contains an N-terminal lobe, which is involved in the binding and orientation of an ATP molecule, whereas the C-terminal lobe is responsible for binding to the protein substrate and transferring of the phosphate group. ATP binds to a deep cleft between the two lobes. These similarities suggest that bacterial and eukaryotic STKs share conserved ATP-binding and hydrolysis mechanisms (Janczarek et al., 2018). Additional domains mediate the binding of ligands and/or protein-protein interactions, such as penicillin-binding proteins and serine/threonine kinase associated (PASTA) domains (Krupa and Srinivasan, 2005). A study analyzing *B. subtilis* revealed an interaction between PASTA motifs and peptidoglycan, the ligand of the STK receptor (Shah et al., 2008). Several *in vitro* studies have also demonstrated that PASTA motifs are able to bind β-lactams and peptidoglycan fragments, making STK as a cell membrane receptor that transmits information from the cell wall state to the phosphorylation target (Maestro et al., 2011; Mir et al., 2011). Importantly, STKs with PASTA motifs play a major role in the regulation of bacterial cell division and morphogenesis (Pereira et al., 2011). The activation of STKs is thought to be initiated by the binding of these neuropeptide ligands, resulting in dimerization and subsequent autophosphorylation of the cytoplasmic N-terminal kinase domain. This leads to the phosphorylation of downstream target proteins and eventually results in the modulation of transcriptional activity. This process has been confirmed in a study of *Mycobacterium tuberculosis* and *Staphylococcus aureus* (Barthe et al., 2010; Ohlsen and Donat, 2010). A topological analysis predicted PknB, the serine/threonine protein kinase of *S. mutans*, as a transmembrane protein with a catalytic domain in the cytoplasm and a C-terminal domain located extracellularly (**Figure 2**). Three PASTA domains are located at the C terminus (Hussain et al., 2006).

### Serine/Threonine Phosphatases

The serine/threonine phosphatase system has long been considered as an exclusive PTM in eukaryotes for a long time (Bakal and Davies, 2000), and the first reported bacterial example was the *E. coli* tricarboxylic acid cycle enzyme isocitrate dehydrogenase (IDH) (Garnak and Reeves, 1979). Most enzymes with serine/threonine phosphatase (STP) activity are members of two structurally different families, PPMs and PPPs. A large number of identified and biochemically characterized STPs belong to the PPM family (Shi et al., 1998; Kennelly, 2002). They share a common catalytic domain consisting of 9-11 signature sequence motifs containing eight conserved amino acid residues and eight invariant residues (one Asp in motifs 1 and 2, Thr in motif 4, Gly in motifs 5 and 6, Asp and Gly in motif 8, and Asp in motif 11) (Kennelly, 2002; Zhang et al., 2004; Zhang and Shi, 2004). The phosphatase activity of STPs in the PPM family is dependent on mental status (Kamada et al., 2020). The conserved STP structure is highly parallel to the human PP2C phosphatase. The active site was surrounded by a central β-sandwich, with a pair of α-helices in the flank, and a binuclear metal center is located within the channel of the β-sandwich, and two metal ions located at the base of the cleft (Shi, 2009; Pereira et al., 2011). There are some key differences between the structure of STPs and the human PP2C family, such as Mn^2+^ and Mg^2+^; a structural analysis revealed that bacterial enzymes have a third metal ion bound within the catalytic core (Pullen et al., 2004; Bellinzoni et al., 2007; Schlicker et al., 2008). Another difference is the lack of his62 residue in the bacterial structure, which has been shown to function as an acid that splits the phosphate oxygen bond in human PP2C (Das et al., 1996). The most remarkable structural difference corresponds to the flap subdomain. In bacteria, this region is located further away from the active site. As a mobile element, it may facilitate binding and turnover of the substrates, and introduce the specificity to the dephosphorylation of the substrates (Pereira et al., 2011). Most serine/threonine phosphatases of the PPP family have dual specificity and can also dephosphorylate phosphor-histidine and phosphor-tyrosine residues (Wright and Ulijasz, 2014; Chen et al., 2017). PppL of *S. mutants* was the first reported oral bacterial STP (Banu et al., 2010). However, its structure requires further investigation. The haloacid dehalogenase (HAD) family phosphatase is also widespread in prokaryotes, and it is characterized by a Rossman-like fold with active motif (DxDx[V/T]) (Tribble et al., 2006). The HAD family of phosphatases uses aspartic acid as a nucleophile to form phosphatase intermediates during the phosphoryl transfer process, and absolutely requires divalent ion cofactors (Seifried et al., 2013). SerB of *P. gingivalis* is a well-studied HAD family phosphatase in oral bacteria (**Table 1**). SerB is secreted by *P. gingivalis* and is involved in oral bacteria-host interactions, which will be described in subsequent sections.

**Table 1:** Oral bacterial protein kinases and phosphatases.

Organism	Kinase or phosphatase	Substrates	Function	Ref
*S. mutans*	PknB^a^, PppL^b^ (Ser/Thr)	–	cell wall biosynthesis, cell transformation, biofilm formation, environmental stress tolerance, bacterial cariogenicity, bacteriocins production, regulation of Smu2146c, VicRK, and ComDE	(Hussain et al., 2006; Banu et al., 2010)
*S. mutans*	PknB^a^ (Ser/Thr)	–	H_2_O_2_ resistance of *S. mutants* in the interspecies competition with *Streptococcus sanguinis*	(Zhu and Kreth, 2010)
*P. gingivalis*	Ptk1^a^, Ltp1^b^ (Tyr)	EpsD, CdhR	*P. gingivalis*-*S. gordonii* community formation, bacterial virulence, EPS production, bacterial virulence	(Maeda et al., 2008; Wright et al., 2014; Liu et al., 2017)
*P. gingivalis*	Ptk1^a^, Ltp1^b^ (Tyr)	UDP-acetylmannosamine dehydrogenase and UDP-glucose dehydrogenase	*P. gingivalis*-*S. gordonii* community formation and EPS production	(Maeda et al., 2008; Liu et al., 2017)
*P. gingivalis*	Ptk1^a^, Ltp1^b^ (Tyr)	PTEN	migration, proliferation, and epithelial mesenchymal transition of epithelial cells	(Liu et al., 2021)
*P. gingivalis*	Php1^b^	Ptk1	EPS production and community development with *S. gordonii* under nutrient-depleted conditions	(Jung et al., 2019)
*P. gingivalis*	SerB^b^ (Ser)	Cofilin	bacterial invasion efficiency, bacterial internalization, and survival	(Moffatt et al., 2012)
*P. gingivalis*	SerB^b^ (Ser)	GAPDH	bacterial invasion efficiency, rearrangement of microtubules to the cell surface	(Tribble et al., 2006)
*P. gingivalis*	SerB^b^ (Ser)	NF-κB RelA/p65	host inflammatory pathways and innate immunity repression, inhibition of IL-8 secretion	(Takeuchi et al., 2013)
*P. gingivalis*	UbK1^a^ (Ubiquitous)	RprY	transcriptional function	(Perpich et al., 2021)

a Kinase.

b Phosphatase.

## The Function of Oral Bacterial Tyrosine and Serine/Threonine Kinases and Phosphatases

### Tyrosine and Serine/Threonine Kinases and Phosphatases in Bacterial Metabolism and Virulence

The first identification and characterization of tyrosine phosphorylation in bacteria appeared in 1996, when Bertrand Duclos et al. revealed the autophosphorylation of tyrosine residues in *Acinetobacter johnsonii* (Duclos et al., 1996). Increasing evidence has demonstrated that tyrosine phosphorylation is crucial for bacterial survival and pathogenicity (Ge and Shan, 2011; Whitmore and Lamont, 2012). Studies have shown that tyrosine phosphorylation is involved in the biosynthesis and export of extracellular polysaccharides (EPS), which are key virulence factors and integral components of biofilm communities (Schwechheimer et al., 2020; Whitfield et al., 2020; Zhuang et al., 2020). Ltp1, the LMW-PTP in *P. gingivalis*, is critical for bacterial virulence, as it helps to regulate various virulence factors at multiple levels. Ltp1 controls EPS production and secretion by regulating the transcriptional activity of genes involved in K-antigen production (PG 0106-0120) and anionic polysaccharide production (PG 0435-0437) (Maeda et al., 2008). Ltp1 can also control the expression of the LuxS enzyme, which is responsible for AI-2 formation, and promote the intake of hemin, thus increasing the toxicity of *P. gingivalis* (Maeda et al., 2008; Rangarajan et al., 2017). More importantly, the secretion and activity of gingipains (Rgp and Kgp) in *P. gingivalis* was regulated by Ltp1 in distinct manner. The secretion efficiency of the Rgp has been positively correlated with the phosphatase activity of Ltp1. In contrast, the dephosphorylated Kgp shows diminished proteolytic activity (Kariu et al., 2017). Consistently, compared with parental strains, the *php1* mutant exhibited less EPS productivity and caused less alveolar bone loss in murine periodontitis models (Jung et al., 2019).

**Figure 4:**
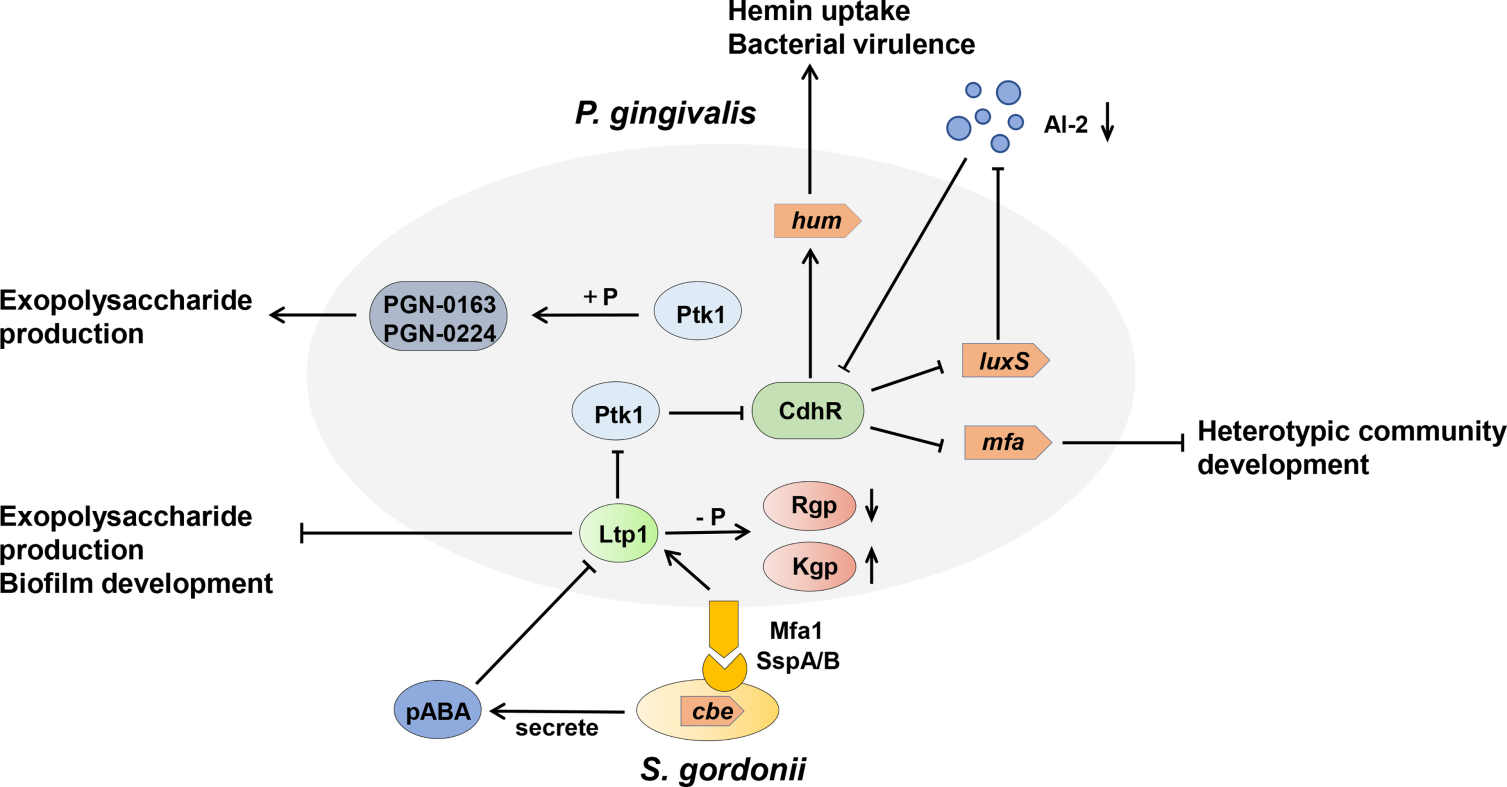
Model of the tyrosine kinase-phosphatase dependent regulatory process governing *Porphyromonas gingivalis* extracellular polysaccharide production, bacterial virulence, and heterotypic community development between *Porphyromonas gingivalis* and *Streptococcus gordonii*. The interactions between *P. gingivalis* and *S. gordonii* resulting from pABA perception and direct contact of *P. gingivalis* Mfa fimbriae with *S. gordonii* Ssp proteins, which can influence Ltp1 activity, thus initiate a cascade of phosphorylation and dephosphrylation events. Ltp1 can decrease the production of exopolysaccharide and dephosphorylate gingipains Rgp and Kgp to affect colony nutrition supply. Ltp1 also dephosphorylates Ptk1 to downregulate its kinase activity, causing the upregulation of CdhR expression. CdhR represses the transcription of *luxS* and *mfa* operons to downregulate the community development of *P. gingivalis* and *S. gordonii* and promotes the transcription of the *hum* operon to increase the hum uptake and virulence of *P. gingivalis*. Lower AI-2 levels cause upregulation of CdhR and constrain the development of a heterotypic community. Conversely, protein kinase Ptk1 uses its enzyme activity to increase the production of exopolysaccharides.

Both Ltp1 and Php1 can be phosphorylated by the tyrosine kinase Ptk1, which is also required for EPS production by *P. gingivalis* (Wright et al., 2014). The 159 and 161 tyrosine residues of Php1 can be phosphorylated by Ptk1, and the 161-residue phosphorylation may indicate a specific regulatory mechanism in *P. gingivalis* (Jung et al., 2019). Interestingly, Ptk1 is also a substrate of Ltp1 and Php1 (Liu et al., 2017; Jung et al., 2019). These results indicated that reversible tyrosine phosphorylation of *P. gingivalis* is tightly orchestrated by the activity of tyrosine kinase (Ptk1) and tyrosine phosphatases (Ltp1 and Php1), allowing the bacteria to sense and respond to perturbations in the environment (**Figure 4**). Further evidence has been derived from high-throughput transposon sequencing has been used to screen the fitness of gene mutants involved in epithelial colonization in a murine abscess model (Miller et al., 2017). Either *php1* or *ptk1* mutant showed reduced fitness in the epithelial colonization model. Thus, the Ptk1-Php1 axis may be prompt the interaction of *P. gingivalis* with host epithelial barriers, functioning as a potential regulator of pathogen colonization and virulence (Miller et al., 2017). UbK1 in *P*. *gingivalis* can also exert its pathogenic function. Specifically, RprY, an orphan two-component system response regulator, can be phosphorylated by UbK1 on Y41 residue, affecting its transcriptional function (Shen et al., 2020; Perpich et al., 2021). The UbK in *S. mutans* has been reported associated with cell morphology and biofilm development (Bitoun et al., 2014).

STK is also essential for bacterial survival and is related to oral biofilm formation related to the oral bacterial. *S. mutans* is a major etiologic agent in dental caries, primarily because of its ability to form biofilms on the tooth surface and to ferment a variety of carbohydrates to produce organic acids (Giacaman, 2018). STK and STP systems play a pivotal role in the pathogenicity of *S. mutans* (Banu et al., 2010). *S. mutans* possesses a STK, PknB. The *pknB* mutant presented a transformation defect, reduced biofilm formation, and reduced the microbial growth rate in culture medium at pH 5.0 and sensitivity to low pH, as well as oxidative and osmotic stress (van der Ploeg, 2005; Hussain et al., 2006). A whole-genome transcriptome analysis revealed that the *pknB* mutant exhibited downregulation of *SMU.1895c* and *SMU.1896c*, which are involved in bacteriocin production (van der Ploeg, 2005). The STP of *S. mutans* is encoded by the *pppL* gene located immediately downstream of *pknB*. The mutant of *pppL* and *pknB pppL* double mutants displayed reduced biofilm thickness and transformation defects.

### Tyrosine and Serine/Threonine Kinases and Phosphatases in Oral Bacterial Dysbiosis

Oral bacterial dysbiosis is characterized by disruption in bacterial homeostasis, caused by an imbalance in the microflora, changes in composition, and metabolic activities, which contribute to oral diseases, such as dental caries and periodontitis (Lamont et al., 2018). *P. gingivalis* acts as a critical agent by disrupting bacterial homeostasis (Mulhall et al., 2020; Xu et al., 2020). In dental plaque, *P. gingivalis* can accumulate into a heterotypic community with *S. gordonii* and utilize physiological support, while the heterotypic colonies are more virulent than *P. gingivalis* mono-species infections (Hajishengallis and Lamont, 2016; Jung et al., 2019).The mechanism of *P. gingivalis* accumulation in the *S. gordonii* matrix is due to the metabolite, 4-amino benzoate (pABA), and direct contact between *P. gingivalis* and *S. gordonii*, which is stringently regulated by the Ltp1-Ptk1 and Php1-Ptk1 axes of *P. gingivalis* (Whitmore and Lamont, 2012; Wright et al., 2014; Lamont et al., 2018; Jung et al., 2019). Ltp1 can inhibit the development of *P. gingivalis* and *S. gordonii* communities at the phenotypic level (Maeda et al., 2008). The mechanism describing how Ltp1 regulates this process was further elucidated. The results showed that Ltp1 upregulated and participated in the interspecies signal transmission after contact with streptococcal SspA or SspB surface proteins. The elevated Ltp1 resulted in dephosphorylation and inactivation of Ptk1, thus increasing the expression of community development and hemin regulator (CdhR) and suppressing the transcription of *mfa1*, which limits the development of heterotypic communities (Chawla et al., 2010). In turn, pABA secreted by *S. gordonii* could suppress the activity of Ltp1 and reverse this signaling transduction through the Ltp1-Ptk1 axis.

Ptk1 activity also converges on expression of the other fimbriae encoding genes (*fimA*). Therefore, we can speculate that Ltp1-Ptk1 affects the oral bacterial homeostasis and dysbiosis by regulating the expression of *P. gingivalis* fimbriae in a spatio-temporal dependent manner. The cognate kinase Php1-Ptk1 axis of *P. gingivalis* also participates in oral bacterial homeostasis and dysbiosis regulation *via* distinct mechanisms. Jung et al. demonstrated that *PhpP* mutants showed diminished heterotypic communities of *P. gingivalis* and *S. gordonii*, but had no significant effect on intraspecific communication of *P. gingivalis* (Jung et al., 2019). Php1 can also dephosphorylate Ptk1, however, the activity of Php1 is resistant to the effect of pABA secreted by *S. gordonii*. Thus, the specific mechanism by which Php1-Ptk1 regulates the heterotypic community requires further investigation.

In addition, *Streptococcus sanguinis*, an early colonizing bacterium in dental biofilm, antagonizes other streptococcus colonization and growth by secreting the virulence factor H_2_O_2_.Studies have shown that serine/threonine kinase PknB secreted by *S. mutans* plays a role in its tolerance to H_2_O_2_, which helps *S. mutans* adapt to ecological pressure and interspecific competition with *S. sanguinis* (Zhu and Kreth, 2010).

### Effect of Tyrosine and Serine/Threonine Phosphatases on Oral Bacteria-Host Interaction

**Figure 5:**
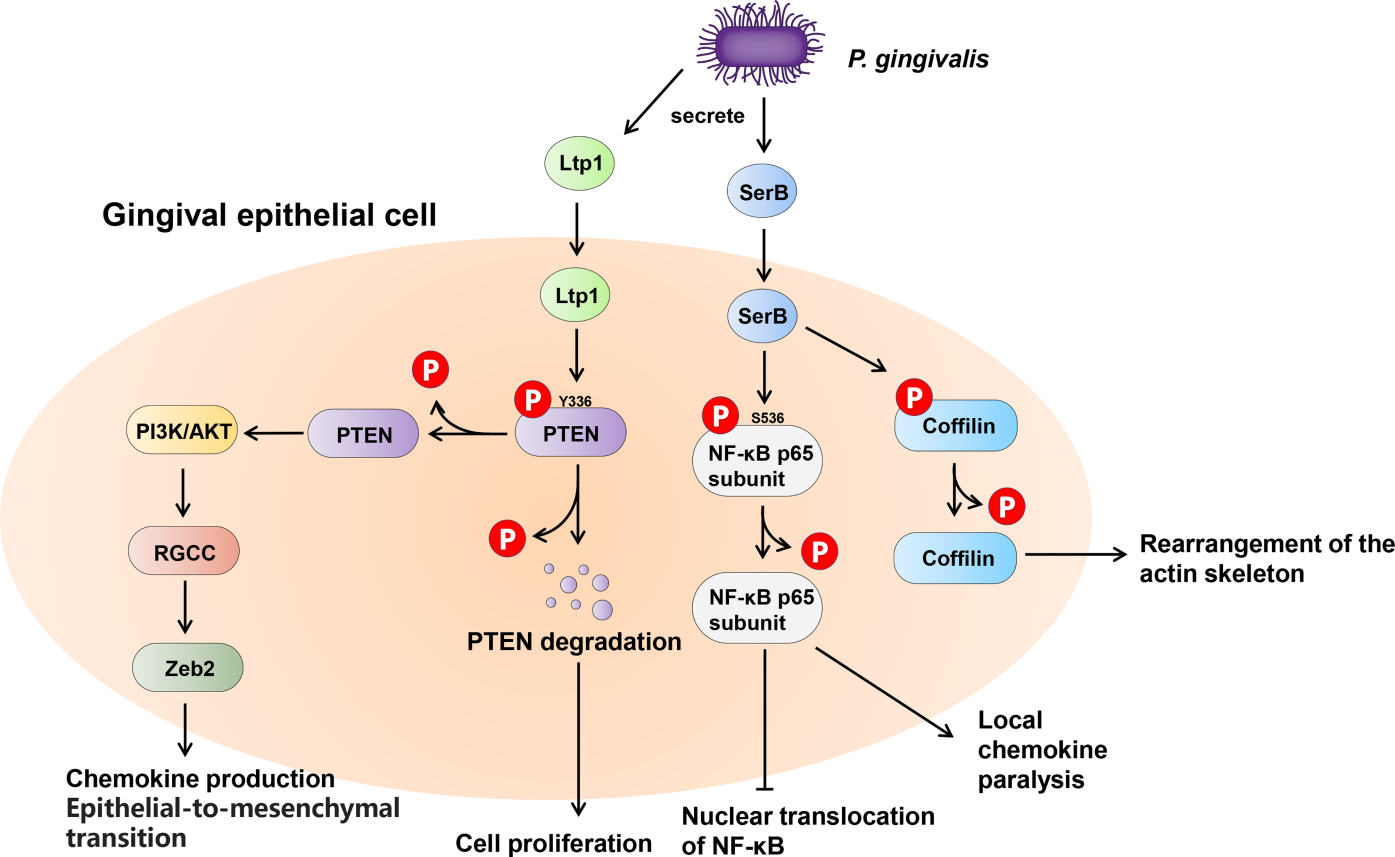
Schematic of the impact of phosphatases secreted by *Porphyromonas gingivalis* within the gingival epithelial cells. *P. gingivalis* secretes two phosphatases, tyrosine phosphatase Ltp1 and serine phosphatase SerB. Upon contacting the gingival epithelial cell, Ltp1 enters the cell to dephosphorylate PTEN, causing proteasomal degradation. Lower PTEN levels promote the PI3K/AKT pathway to upregulate RGCC and Zeb2. SerB dephosphorylates NF-κB and Cofilin to preserve the virulence of *P. gingivalis* and maximize the intracellular invasion of bacteria.

Many bacteria exert their virulence by invading host cells, and the internalization and intracellular survival of bacteria are essential to their pathogenicity (Lewis et al., 2016). Lamont et al. first reported that *P. gingivalis* can invade primary cultures of gingival epithelial cells (Lamont et al., 1995). Mounting evidence supports this finding, and a series of discoveries have since demonstrated the pivotal role of tyrosine and serine/threonine phosphatases in this process (Moffatt et al., 2012; Takeuchi and Amano, 2021). The most common example of the participation of serine/threonine phosphatases in oral bacteria-host interaction is SerB in *P. gingivalis* (**Figure 5**). SerB can be released into host cells and directly interact with host cytoplasmic phosphoproteins, facilitating bacterial internalization (Tribble et al., 2006). The existence of SerB can ensure the invasion of host cells to the greatest extent, as SerB dephosphorylates actin cofilin, an actin depolymerizing host protein, affecting the expression of genes involved in the regulation of actin cytoskeleton dynamics and cytokine secretion (Bainbridge et al., 2010; Woo et al., 2019). Furthermore, SerB can also dephosphorylate the S536 site of NF-κB p65 subunit to prevent nuclear translocation of NF-κB. This process antagonizes the production of interleukin-8 (IL-8), leading to local chemokine paralysis (Takeuchi et al., 2013). Compared to parental strains, the SerB mutant resulted in high levels of neutrophil recruitment to gingival tissue and decreased alveolar bone destruction at both the horizontal and interproximal levels (Bainbridge et al., 2010). In summary, SerB can promote bacterial invasion of the host, allowing it to continue to exert its full pathogenic potential. Interestingly, it has very recently been reported that the tyrosine phosphatase (Ltp1) can also be secreted by *P. gingivalis* and appears in both the cytoplasm and nucleus of gingival epithelial cells (Liu et al., 2021). The secreted Ltp1 can bind to phosphatase and tensin homolog (PTEN) and dephosphorylate its Y336 residue, resulting in the degradation of PTEN. PTEN is a classic negative regulator of phosphoinositide 3-kinases/protein kinase B (PI3K/Akt). Thus, the inhibition of PTEN by Ltp1 could further activate PI3K/Akt and its downstream regulator of the cell cycle (RGCC), promoting the migration, proliferation and epithelial mesenchymal transition of epithelial cells (Liu et al., 2021) (**Figure 5**).

## Conclusion and Perspectives

The phosphorylation system has long been considered an important signal transduction system in eukaryotes, and in recent decades, the function of kinases and phosphatases in prokaryotes has been gradually revealed. Yet, based on the gene homologues of bacteria, there are still many putative kinases and phosphatases that have not been studied (**Table 2**). The rising prevalence of antibiotic-resistant bacteria is driving research toward novel targets. With the advent of phosphor-proteomics, more phosphorylation proteins and sites can be discovered to expand the phosphorylation regulatory network (Misra et al., 2011; Mijakovic and Macek, 2012; Bäsell et al., 2014; Yagüe et al., 2019). In the future, more experiments are needed to verify the specific functions of kinases and phosphatases in oral bacteria physiology and pathogenicity, clarify mechanisms between bacteria and the host, and identify potential drug targets to treat infection, immune responses, and diseases.

**Table 2:** Tyrosine and serine/threonine kinases and phosphatases in *S. mutants* and *P. gingivalis*.

Bacteria	Gene ID	Symbol	Function
*S. mutants UA159*	*SMU_483*	*PppL*	PPM family protein phosphatase (putative)
	*SMU_484*	*PknB*	Serine/threonine protein kinase
	*SMU_65*		Low molecular weight protein-tyrosine phosphatase (putative)
	*SMU_646*		HAD family phosphatase (putative)
	*SMU_754*		Serine kinase/phosphatase (putative)
	*SMU_1269*		Phosphoserine phosphatase (putative)
	*SMU_1747c*		HAD family phosphatase (putative)
	*SMU_1802c*		HAD family phosphatase (putative)
*P. gingivalis* ATCC 33277	*PGN_1524*	*Ptk1*	Tyrosine kinase
	*PGN_0491*	*Ltp1*	Low molecular weight protein-tyrosine phosphatase
	*PGN_1525*	*Php1*	PHP family tyrosine phosphatase
	*PGN_0662*	*SerB*	Serine/threonine protein phosphatase
	*PGN_1020*	*Ubk1*	Ubiquitous bacterial kinase
	*PGN_1267*		Phosphoserine phosphatase (putative)

## Author Contributions

LR, DS and CL drafted the manuscript. CL and YD edited and added valuable insights into the manuscript. All authors approved the final manuscript and agreed to be accountable for all aspects of the work.

## Funding

This study was supported by the National Natural Science Foundation (grant number 82071121 to YD).

## Conflict of Interest

The authors declare that the research was conducted in the absence of any commercial or financial relationships that could be construed as a potential conflict of interest.

## Publisher’s Note

All claims expressed in this article are solely those of the authors and do not necessarily represent those of their affiliated organizations, or those of the publisher, the editors and the reviewers. Any product that may be evaluated in this article, or claim that may be made by its manufacturer, is not guaranteed or endorsed by the publisher.

## Abbreviations

PTMs, post-translational modifications; *M. xanthus*, *Myxococcus xanthus*; *S. pneumoniae*, *Streptococcus pneumoniae;* STKs, eukaryotic serine/threonine kinases; *P. gingivalis*, *Porphyromonas gingivalis*; BY kinases, bacterial tyrosine kinases; UbK, ubiquitous bacterial kinase; *S. gordonii, Streptococcus gordonii; B. subtilis*, *Bacillus subtilis*; *E. coli*, *Escherichia coli*; *S. mutans*, *Streptococcus mutans*; Tyr kinases, tyrosine kinases; LMW-PTPs, low-molecular-weight protein tyrosine phosphatases; PTPs, eukaryotic like phosphatases; PHPs, polymerase–histidinol phosphatases; *M. tuberculosis*, *Mycobacterium tuberculosis*; PASTA, penicillin binding proteins and serine/threonine kinase associated; STPs, serine/threonine phosphatases; PPMs, metal-dependent phosphatases; PPPs, phosphoprotein phosphatases; HAD, haloacid dehalogenase; EPS, extracellular polysaccharide; NF-κB, nuclear factor-kappa B; *S. sanguinis*, *Streptococcus sanguinis.*
